# Cryogenics free production of hyperpolarized ^129^Xe and ^83^Kr for biomedical MRI applications^☆^

**DOI:** 10.1016/j.jmr.2013.09.008

**Published:** 2013-12

**Authors:** Theodore Hughes-Riley, Joseph S. Six, David M.L. Lilburn, Karl F. Stupic, Alan C. Dorkes, Dominick E. Shaw, Galina E. Pavlovskaya, Thomas Meersmann

**Affiliations:** a Sir Peter Mansfield Magnetic Resonance Centre, School of Medicine, University of Nottingham, Nottingham NG7 2RD, United Kingdom; b Sir Peter Mansfield Magnetic Resonance Centre, School of Physics and Astronomy, University of Nottingham, Nottingham NG7 2RD, United Kingdom; c Nottingham Respiratory Research Unit, University of Nottingham, Nottingham NG5 1PB, United Kingdom

**Keywords:** Xenon-129, Xe-129, Krypton-83, Kr-83, Hyperpolarization, Spin-exchange optical pumping, Cryogenic separation, Pulmonary MRI, Lung imaging, Oxygen partial pressure, *T*_1_ relaxation

## Abstract

As an alternative to cryogenic gas handling, hyperpolarized (hp) gas mixtures were extracted directly from the spin exchange optical pumping (SEOP) process through expansion followed by compression to ambient pressure for biomedical MRI applications. The omission of cryogenic gas separation generally requires the usage of high xenon or krypton concentrations at low SEOP gas pressures to generate hp ^129^Xe or hp ^83^Kr with sufficient MR signal intensity for imaging applications. Two different extraction schemes for the hp gasses were explored with focus on the preservation of the nuclear spin polarization. It was found that an extraction scheme based on an inflatable, pressure controlled balloon is sufficient for hp ^129^Xe handling, while ^83^Kr can efficiently be extracted through a single cycle piston pump. The extraction methods were tested for *ex vivo* MRI applications with excised rat lungs. Precise mixing of the hp gases with oxygen, which may be of interest for potential *in vivo* applications, was accomplished during the extraction process using a piston pump. The ^83^Kr bulk gas phase *T*_1_ relaxation in the mixtures containing more than approximately 1% O_2_ was found to be slower than that of ^129^Xe in corresponding mixtures. The experimental setup also facilitated ^129^Xe *T*_1_ relaxation measurements as a function of O_2_ concentration within excised lungs.

## Highlights

• Production of hyperpolarized (hp) ^129^Xe and hp ^83^Kr without cryogenic processing.
• Single step extraction, transfer, and compression of hp gas after optical pumping.
• Methodology is applied to obtain hp ^129^Xe and hp ^83^Kr MR images of excised rat lungs.
• Precise mixing of hp gases with oxygen after extraction and before compression.
• Oxygen dependent *T*_1_ relaxation in bulk gas phase and in excised rat lungs.

## Graphical abstract

**Figure:**
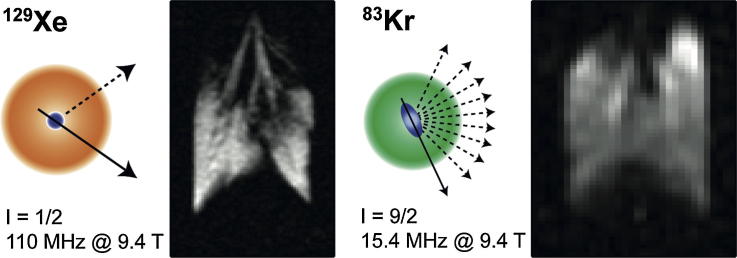

## 1 Introduction

Biomedical noble gas MRI applications require high signal intensities that can be obtained through the hyperpolarization of the nuclear spin state. Hyperpolarization is generated through spin exchange optical pumping (SEOP) using high power laser irradiation of alkali metal vapor (typically rubidium) in the presence of the noble gas isotope [1–10]. Although nuclear spin polarization levels around 50% can now be produced for ^129^Xe at rates of liters per hour [7,11], the high costs involved with the reliable hyperpolarized (hp) ^129^Xe production may impede its widespread MRI usage. Developments, such as small and transportable hyperpolarizer units [12], that make ^129^Xe MRI more affordable would help to proliferate clinical or pre-clinical usage and therefore foster further exploration of hp ^129^Xe as a pathophysiological biomarker. Furthermore, a new approach is needed for hp ^83^Kr MRI where the quadrupolar nature of the isotope causes challenges for the production process. Hp ^83^Kr applications in pulmonary research were thus far limited to low resolution MRI [13,14] and to spatially unresolved relaxation measurements in rat lungs [15].

The objective of this work was to omit cryogenic separation in the hp noble gas production process for pulmonary MRI. The ‘cryogenics-free’ concept [16] is beneficial for reducing the complexity, and therefore the costs, of the hp ^129^Xe production. Furthermore, this concept is crucial for biomedical hp ^83^Kr MRI since quadrupolar relaxation causes the loss of the hyperpolarized spin state during cryogenic separation. The streamlined hp ^129^Xe and hp ^83^Kr production procedure without cryogenic gas separation was tested in applications for MRI of excised rat lungs. The developmental work utilized *ex vivo* lungs in order to simplify experimental and regulatory procedures but the general concepts will be extendible to *in vivo* MRI.

## 2 Background

### 2.1 Stopped flow SEOP with highly concentrated ^129^Xe or ^83^Kr at low pressures

Low xenon concentrations are typically used for ^129^Xe SEOP because a high density of this noble gas is detrimental to the process. The noble gas is usually diluted to 1–5% in mixtures with molecular nitrogen or helium (i.e. ^4^He). In the case of helium as the diluting gas, approximately 2–5% N_2_ are added in the mixture to ensure radiation quenching [10,17]. The low xenon density in the SEOP gas mixture enables high spin polarization to be generated and values with *P* > 60% have been reported [6–8]. However, the method necessitates cryogenic separation after SEOP with hp xenon accumulation in the frozen state at cryogenic temperatures (typically 77 K) and the removal of all other gases of the mixture through evacuation [18]. In analogy to ^129^Xe SEOP, a low concentration of krypton is crucial for efficient SEOP of ^83^Kr. Despite the quadrupolar driven ^83^Kr *T*_1_ relaxation, a high spin polarization of *P* = 26% in a gas mixture of 5% krypton and 95% N_2_ was obtained in stopped flow SEOP [10]. Unfortunately for hp ^83^Kr MRI, there is currently no practical method to separate or concentrate hp ^83^Kr from the gas mixture without substantial depolarization of its nuclear spin state. Fast quadrupolar driven *T*_1_ relaxation in the condensed state [19,20] prevents cryogenic separation of this isotope and the production process has to be realized without gas separation.

The need for cryogenic separation is diminished if concentrated noble gas mixtures are used in low pressure SEOP. The associated detrimental effects of high xenon or krypton densities can partially be alleviated by low SEOP gas pressure [10,21,22]. However, the pressure broadening of the alkali metal D_1_ transition is also reduced with lower SEOP pressures and therefore narrow laser linewidth are beneficial. Note that line narrowed diode array lasers with high power output are becoming increasingly available at affordable costs [23–25]. In a recent study, the pressure dependence of the SEOP process under stopped flow conditions was explored for various gas mixtures using a line-narrowed laser (i.e. 0.23 nm FWHM at 794.7 nm) [10].

**Fig. 1:**
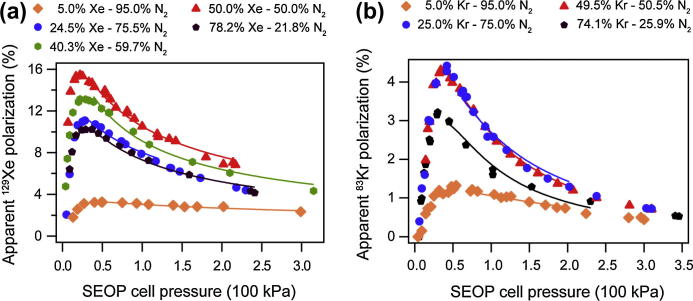
Apparent spin polarization as a function of SEOP pressure for ^129^Xe and ^83^Kr. Data taken from reference [10] but reprocessed to display apparent polarization. (a) ^129^Xe apparent spin polarization (polarization accounting for buffer gas dilution) for five different gas mixtures. Data for the 40.3% Xe–59.7% N_2_ mixture are original to this work. Data analysis were preformed as described previously [10]. (b) ^83^Kr apparent spin polarization *P_app_* for different gas mixtures. See legend for icon explanation.

Using stopped flow SEOP, the highest ^129^Xe polarization was found at pressures between 22 and 46 kPa depending on the mixture used as shown in Fig. 1. Similarly, the highest ^83^Kr polarization value for the various gas mixtures were found at a pressure range between 30 and 54 kPa. In stopped flow SEOP, the gas mixture remains in the SEOP cell until a (near) steady state polarization is obtained, thus maximizing the obtained spin polarization. Note that the stopped flow mode is crucial for the production of hp ^83^Kr for MRI applications. Furthermore, stopped flow SEOP opens up the possibility for a single extraction–compression cycle for the hp noble gases.

### 2.2 The apparent polarization *P_app_*

In order to simplify comparison of the MR signal expected form diluted hp gas mixtures with that of concentrated hp ^129^Xe, the apparent polarization, *P_app_*, was defined for hp gas mixtures:

(1) $$P_{\mathrm{app}}=P\cdot \frac{\left[\text{NG}\right]}{\sum_{i}[M_{i}]}$$

where the scaling of the spin polarization, *P*, is taken into account through the noble gas (number) density, [NG], divided by the overall (number) density of all components *M_i_* in the mixture [10]. This definition is useful because *P_app_* allows for easy comparison of the signal intensities from diluted hp noble gas mixtures – i.e. a dilute mixture with *P_app_* = 10% results in the same NMR signal intensity as that of a pure hp noble gas with *P* = 10%.

In the previous work, using 23 W of incident laser power, the highest apparent polarizations for hp ^83^Kr were found with the $P_{\mathrm{app}}=4.4\pm 0.5\%$ for the 25% krypton–75% N_2_ mixture and $P_{\mathrm{app}}=4.3\pm 0.5\%$ for the 50% krypton–50% N_2_ gas mixture. Higher and lower krypton concentration quickly leads to reduced apparent polarizations as shown in Fig. 1. Similarly, the highest ^129^Xe polarization was found for the 50% xenon–50% N_2_ mixture with $P_{\mathrm{app}}=15.5\pm 1.9\%$.

## 3 Extraction of the hp gas mixture from low pressure SEOP cells

**Fig. 2:**
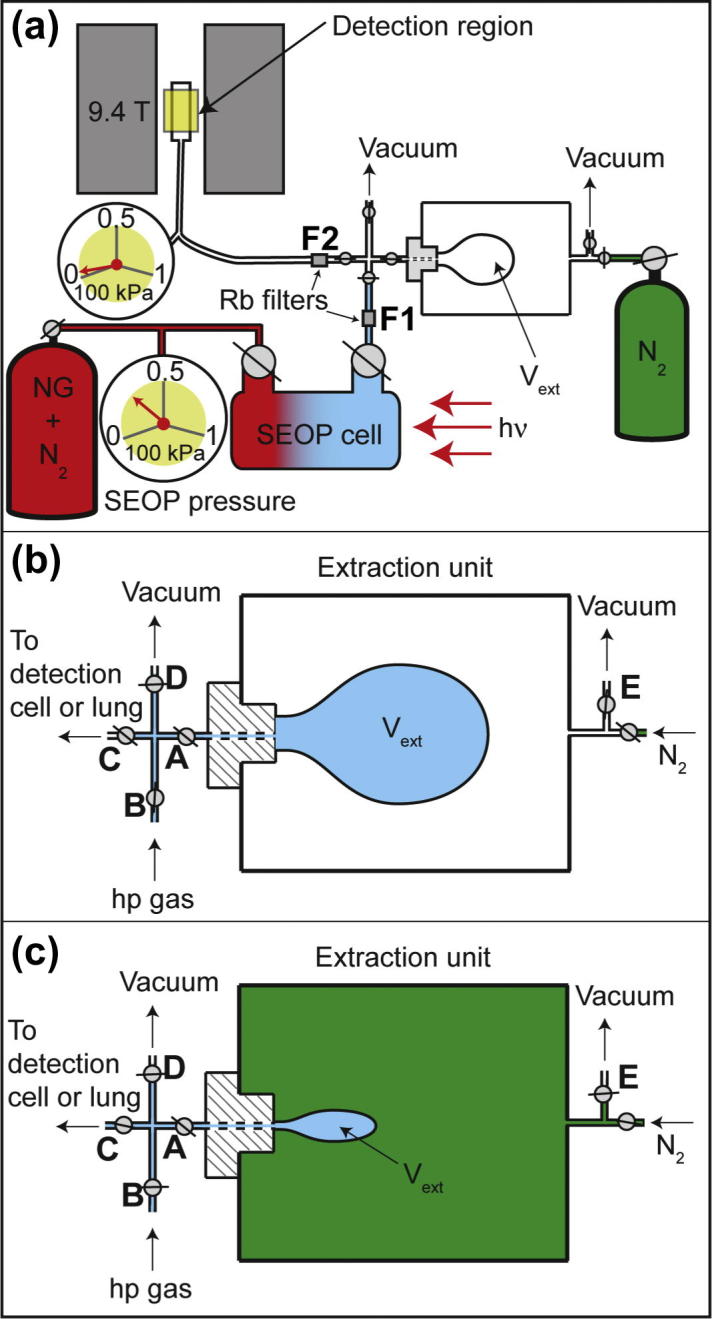
Production and delivery of hp noble gas using Extraction Scheme 1. (a) Noble gas–nitrogen gas mixtures are hyperpolarized in the SEOP cell. In the ‘Baseline Scheme’ measurements the extraction unit is closed to the SEOP cell and hp noble gas–nitrogen mixture is transferred to a glass detection cell via pressure equalization. Note that the Rb filters (F1 and F2) were only used for Rb vapor analysis described in Section 4.3 (b and c) Outline of device used in ‘Extraction Scheme 1’. Internal volume of the latex balloon serves as volume *V_ext_*. Hp gas mixture is transferred from SEOP cell into *V_ext_* as shown in (b). The main chamber of the extraction unit is pressurized with N_2_ causing the balloon to collapse after closing of valve A as shown in (c). The hp gas is then redirected through valves A and C towards its intended target.

An apparent ^129^Xe polarization of *P_app_* = 15.5% as shown in Fig. 1 is sufficiently high to consider the cryogenics free hp ^129^Xe production for biomedical MRI applications. However, the cryogenic process does not only facilitate gas separation, it usually also enables gas transport from the SEOP cell to a small volume cold finger during the freezing phase. Subsequent sublimation of the frozen hp ^129^Xe allows for recompression of the hp gas to ambient pressure or above. If this step is omitted, some other means of hp gas transportation needs to be instituted for low pressure SEOP. For simple polarization measurements the hp gas can be transferred through expansion from the SEOP cell through transfer tubing into a pre-evacuated sample cell for NMR detection at low pressures (Fig. 2). This method was used in this work to provide baseline data and is therefore dubbed ‘Baseline Scheme’. However, for biomedical applications, such as lung MRI in an ambient pressure environment, the hp gas is required to be compressed before usage. This task, by itself, is not new as metastability exchange optical pumping (MEOP) of ^3^He typically takes place at low pressures in batch mode and a number of techniques to recompress hp ^3^He have been successful [26–28]. Further, a diaphragm pump was used by Imai et al. [21] to recompress hp ^129^Xe for low pressure SEOP with reported loses as low as 1/10 of the polarization value.

This work focused on hp gas extraction in a single expansion–compression cycle. The transport from the low pressure SEOP cell was accomplished by expansion into a large volume of a collapsible container. The volume *V_ext_* of the respective gas expansion chamber was required to be much larger than that of the SEOP cell (*V_SEOP_*) to allow for a rapid transfer of a large portion of the hp gas. The extraction container was then collapsed and its content was pressurized to ambient by the application of external gas pressure. Two designs were explored to facilitate the extraction scheme: Extraction Scheme 1 – The first design used an inflatable balloon and was intended to minimize machining requirements during fabrication and complexity during operation. A latex balloon was used in this to allow for a large volume *V_ext_* and large pressure differential. Extraction Scheme 2 – The second design utilized a gas-operated piston and was more demanding for the manufacturing process because it needed to ensure smooth running but also tight operation of the piston within a cylinder (see Section 6).

Fig. 2 shows the straightforward concept of Extraction Scheme 1 with an inflatable latex balloon as expansion volume. The balloon was contained within a gas tight chamber that could be pressurized or evacuated depending on the required task. The inside of the balloon was connected, via the valves A and B, to the SEOP cell and could take up a high fraction of the hp gas mixture.

During stopped flow SEOP, the interior of the balloon and the surrounding external space were both evacuated causing the balloon to assume a collapsed state due to the elasticity of the latex. The hp gas was then transferred into the balloon while its external volume (i.e. the pressure control chamber) was still connected to the vacuum pump. Following the hp gas transfer, the balloon was compressed above ambient by filling the pressure control chamber with pressurized N_2_ (typically 100–200 kPa above ambient to ensure fast compression). The hp gas was transferred to the pre-evacuated detection cell for the NMR polarization measurement by opening valves A and C. The spin polarization of the hp gas determined from this measurement was approximately the same as the polarization of the inhaled gas for the pulmonary MRI measurements.

**Fig. 3:**
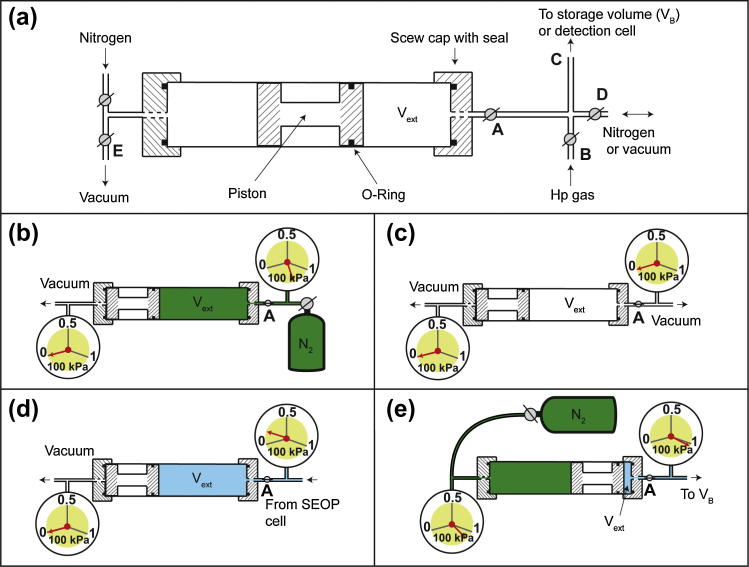
The extraction unit used for Extraction Scheme 2. (a) Diagram of the two-chamber extraction unit with the front chamber (*V_ext_*) to accommodate the hp gas. The chambers are separated by a single piston equipped with an O-ring; the piston is driven by a pressure differential between the two chambers. (b) The piston is moved to the back position through pressurization of *V_ext_* with N_2_ and simultaneous evacuation of the back chamber. (c) *V_ext_* was evacuated to prepare for hp gas extraction from SEOP cell. (d) Hp gas extraction from SEOP cell is complete and *V_ext_* is filled with approximately 6 kPa of hp gas. (e) The volume *V_ext_* is sealed by valve A and the back chamber is pressurized with N_2_ forcing the piston forward thus compressing the hp gas. Once the hp gas in *V_ext_* is above ambient pressure valves A + C are opened and hp gas is injected into a temporary storage container for inhalation.

The second design, sketched in Fig. 3, utilized a pressure driven piston (Extraction Scheme 2). The movable piston sealed two parts of a cylinder, thus it allowed for a variable volume *V_ext_* on one side of the piston while the other side of the piston was used as a pressure control chamber. Like a syringe, the gas extractor could remove the hp gas from the SEOP cell, compress it, and finally ‘inject’ the hp gas into a target system. This setting is reminiscent to the approach used by Rosen and co-workers for hp ^129^Xe [4], however this concept was extended to accommodate the high pressure-differential during hp gas extraction and compression.

## 4 Results and discussion

### 4.1 Hp ^129^Xe extraction

**Fig. 4:**
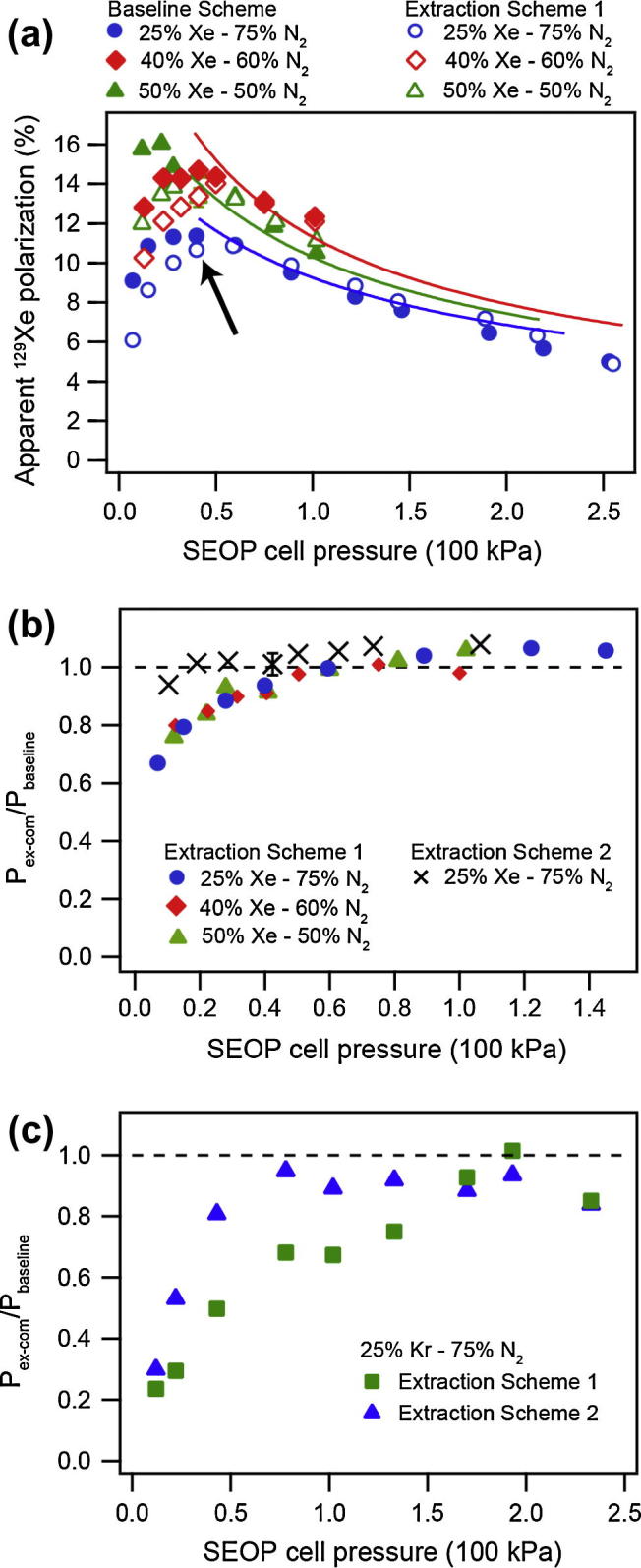
Hp gas polarization using different extraction schemes. (a) ^129^Xe apparent spin polarization for three different gas mixtures with Baseline Scheme and Extraction Scheme 1 (see figure legend for clarity). Solid lines show data analysis from Fig. 1 for comparison with current experimental data. The arrow indicates the SEOP cell pressure used for ^129^Xe imaging experiments. (b) Fraction of the ^129^Xe polarization after the extraction–compression process (*P*_ex-com_/*P*_baseline_) or “polarization survival” as a function of SEOP cell pressure for three different gas mixtures using Extraction Schemes 1 and 2. (c) *P*_ex-com_/*P*_baseline_ of the ^83^Kr polarization as a function of SEOP cell pressure using Extraction Schemes 1 and 2. Polarization losses were greater when Extraction Scheme 1 was used at low pressures (below 150 kPa), making Extraction Scheme 2 a superior choice for ^83^Kr imaging studies.

The apparent spin polarization *P_app_* obtained after hp ^129^Xe transfer with Extraction Scheme 1 is shown in Fig. 4a as a function of SEOP pressure for various SEOP mixtures (open symbols). The apparent polarization *P_app_* of hp ^129^Xe transferred directly from the SEOP cell into the NMR detection cell served as baseline data, also shown in Fig. 4a (filled symbols). At SEOP pressure above approximately 50 kPa little difference was found in the spin polarization *P_app_* between baseline data and Extraction Scheme 1. Polarization losses below this pressure are visualized in Fig. 4b where the Extraction Scheme 1 polarization data was normalized by the respective baseline values (filled symbols). The normalized data demonstrates that the losses occurring below 50 kPa were gas mixture independent.^3^

Fig. 4b also displays data using Extraction Scheme 2 (crosses) and it can be seen that polarization losses appeared only for SEOP pressures below 0.2 kPa.

Both devices (Extraction Schemes 1 and 2) allowed for cryogenics free hp ^129^Xe extraction at acceptable losses in the polarization at experimentally useful SEOP pressure conditions. Extraction Scheme 2 was slightly advantageous at lower pressures over the balloon based Extraction Scheme 1 probably because it accommodated the hp gas transfer more rapidly and it therefore reduced the overall relaxation during the transfer. Unlike Expansion Scheme 1, where the expanding gas had to perform work against the surface tension of the balloon, the piston in Extraction Scheme 2 was already pushed into its ‘backward’ position before the gas transfer. Therefore, the hp ^129^Xe expanded directly into the evacuated volume *V_ext_*, a process that was faster than Extraction Scheme 1 where time was required to inflate the balloon. Nevertheless, Fig. 4 shows that $P_{\mathrm{app}}\approx 14\%$ were obtained with Extraction Scheme 1.

Hp gas extraction with the Extraction Scheme 1 took approximately 5 s until a pressure of about 40–150 kPa, depending on the initial SEOP pressure, was reached. Compression to above atmospheric pressure was accomplished within 6 s and the gas was transferred into the NMR detection cell 15 s after commencement of the extraction process. Similarly, using Extraction Scheme 2, the gas was allowed to expand until a pressure of about 6–13 kPa was measured leading to about 3/4 of the hp gas to be transferred into the cylinder. Compressing the hp gas to above ambient pressures took 3 s and the gas transfer into the detection cell was complete within 10 s after the initiation of the extraction process. The fraction of hp gas transferred was less than would be expected given the volumes of the SEOP cell, $V_{\mathrm{ext}}^{\max}$, the initial pressure during SEOP, and the final pressure after extraction. However, the transfer of the hp gas at the remaining small pressure differential towards the end of the extraction process was slow. Prolonged transfer times that allow for a maximized hp gas transfer were found to be detrimental to the overall spin polarization of the final hp gas sample.

**Fig. 5:**
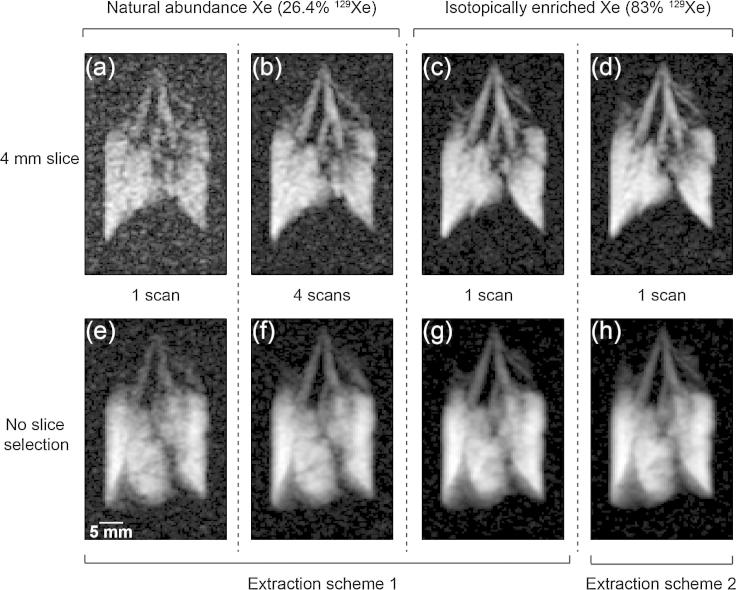
^129^Xe VFA FLASH coronal images of *ex vivo* rat lungs using Extraction Scheme 1 and Extraction Scheme 2. Image size was 128 × 64 with FOV = 46.9 × 30.0 mm^2^. The lungs were ventilated with 4 ml hp ^129^Xe gas mixture. (a) A 4 mm central slice collected in a single acquisition (NEX = 1) using natural abundance xenon and Extraction Scheme 1 (SNR = 8.1). (b) Four image acquisitions (NEX = 4) similar to (a) collected from different inhalation cycles and averaged (SNR = 13.8). (c) A 4 mm central slice collected in a single acquisition (NEX = 1) using isotopically enriched xenon (83% ^129^Xe) and Extraction Scheme 1 (SNR = 24.7). (d) A 4 mm central slice collected using isotopically enriched xenon similar to (c), where Extraction Scheme 2 was used (NEX = 1, SNR = 22.1). There was little noticeable difference between the images of the two Extraction Schemes. (e–h) Non-slice selective images corresponding to (a–d) slice-selective images (SNR_e_ = 19.3; SNR_f_ = 41.2; SNR_g_ = 64.4; SNR_h_ = 81.6) collected as described in Section 6.4.

Using a 40% xenon in nitrogen mixture and an SEOP at pressure of 50 kPa, roughly 18 ml of hp ^129^Xe (with Extraction Scheme 1) with $P_{\mathrm{app}}\approx 14\%$ were obtained (Fig. 4). For the imaging experiments, a 25% xenon mixture was used at 40 kPa leading to a lower polarization of *P_app_* = 10.9 ± 0.1% that was delivered for inhalation to an excised rat lung (see Section 6 for further experimental details). Since this polarization led to excellent image quality shown in Fig. 5, the experiments were not repeated with the 40% mixture. A single, cryogenics free delivery of hp ^129^Xe was used and 4 ml of the hp gas mixture were inhaled by the excised rat lung for each MRI without signal averaging (Fig. 5a, c, d, e, g and h) or for each of the scans when signal averaging was applied (Fig 5b and f). Variable flip angle (VFA) FLASH MRI sequence [29] was applied to utilize the complete hyperpolarized spin state.

Imai et al. had previously demonstrated *in vivo* hp ^129^Xe MRI under continuous flow conditions without cryogen usage. This method allowed for, but also required, many inhalation cycles. However, Fig. 4 demonstrates that cryogenics free, slice selective MRI is feasible within a single scan (number of experiments; NEX = 1) with the extraction schemes presented in this work, at least for *ex vivo* work. Note that the high applied field strength of 9.4 T was not necessarily advantageous for pulmonary hp ^129^Xe MRI due to strong magnetic field inhomogeneities in the heterogeneous medium leading to fast transverse relaxation with $T_{2}^{⁎}$ = 1.77 ± 0.37 ms. *In vivo* application of this method was not explored in this work, however Extraction Scheme 1 was applied to *ex vivo* lung functional studies, including post mortem airway sensitivity to methacholine challenge, published elsewhere [30].

### 4.2 Hp ^83^Kr extraction

Due to quadrupolar relaxation that causes fast depolarization, a rapid gas transfer is crucial for the hp ^83^Kr extraction if polarization losses are to be minimized. Since transfer rate of the hp gas was dependent on the extraction scheme (see discussion in the *Hp ^129^Xe extraction* section) one would expect clear differences in the observed hp ^83^Kr spin polarization between Extraction Scheme 1 and 2. As shown in Fig. 4c, the slower Extraction Scheme 1 lead to substantial polarization losses compared to baseline data at all SEOP pressures below 150 kPa (filled squares). There was a clear advantage of Extraction Scheme 2 (triangles) and approximately 80% of the baseline polarization was recovered with this method at SEOP pressures above 50 kPa. The higher losses in polarization with Extraction Scheme 1 probably occurred not only because of the slower transfer rate but also because of the large surface to volume ratio during the stages when the balloon was partially collapsed.

The baseline scheme applied to the 25% krypton–75% nitrogen mixture after SEOP at 50 kPa lead to a maximum apparent spin polarization of *P_app_* = 4.4% (as shown in Fig. 1) and approximately 80% were recovered with Extraction Scheme 2 leading to $P_{\mathrm{app}}\approx 3.5\%$. For the hp ^83^Kr MRI with natural abundance (11.5%) ^83^Kr shown in Fig. 5a and b the SEOP pressure was kept at a higher pressure around 85 kPa leading to 34 ml of hp gas with $P_{\mathrm{app}}\approx 3.3\%$ through Extraction Scheme 2 (Baseline Scheme $P_{\mathrm{app}}\approx 3.5\%$). An 8 ml quantity of hp ^83^Kr gas mixture was inhaled by the lung from *V_B_* (see Section 6) within 3 s after delivery but the extent of hp ^83^Kr depolarization in this container was not determined. The ^83^Kr polarization was sufficient to produce a coronal, non-slice selective image at about half of the resolution as the corresponding hp ^129^Xe MR images.

**Fig. 6:**
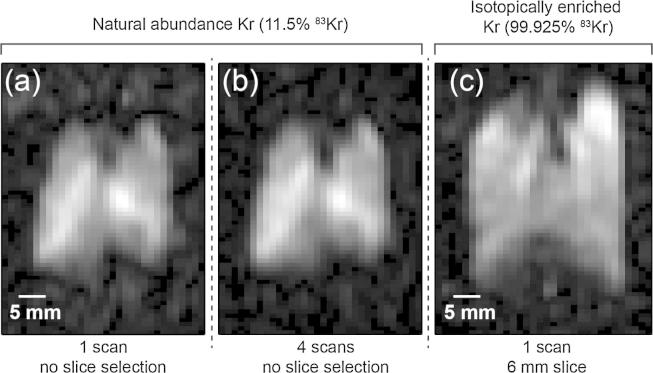
^83^Kr VFA FLASH coronal images of *ex vivo* rat lungs using Extraction Scheme 2. Image size is 64 × 32. The lungs were ventilated with 8 ml hp ^83^Kr gas mixture. (a) A non-slice selective image collected in a single acquisition with FOV = 51.0 × 38.1 mm^2^ (NEX = 1, SNR = 12.5). (b) Four non-slice selective images similar to (a) collected over different inhalation cycles and averaged. (NEX = 4, SNR = 25.4). (c) A 6 mm central slice collected in a single acquisition (NEX = 1) using isotopically enriched krypton (99.925% ^83^Kr) with FOV = 50.9 × 40.7 mm^2^ (SNR = 24.7).

Due to the low natural abundance of ^83^Kr, the resulting MR images were improved drastically using isotopically enriched (i.e. 99.925%) ^83^Kr as shown in Fig. 6c. Isotopically enriched ^83^Kr is quite expensive with approximately € 4000 per liter gas (at 100 kPa) and only a small quantity was available for the experiments. Therefore, mixing of the costly gas with N_2_ was done *in situ* within the SEOP cell and resulted into slightly higher SEOP pressures around 90–100 kPa that produced approximately 40 ml hp gas mixture at ambient pressure with an apparent polarization of $P_{\mathrm{app}}\approx 2.4\%$ after Extraction Scheme 2.

### 4.3 Rubidium vapor

Rubidium metal atoms, forming a solid at ambient temperatures, were present in the vapor phase during SEOP but most of the metal should have been condensed during hp gas transfer within the connecting tubes and the extraction unit. However, the cryogenic-free extraction schemes may raise concerns about physiologically harmful quantities of rubidium vapor that could potentially pass along with the hp gas mixture through the extraction process. To investigate whether physiologically significant pH changes could have been caused by any remaining rubidium vapor in the extracted hp gas mixtures, gas filters were inserted into the transfer lines at two locations (see Fig. 2a). Note, all polarization measurements and MRI reported in this work were obtained without these filters. Filters were used only in separate measurements to serve as a probe for the presence of rubidium.

Filters were tested with hp ^83^Kr production at the associated high SEOP temperatures (170 ± 5 K). After a certain number of cycles the filters were removed and washed with 1.0 ml distilled water. The strongest pH change, +2.5, was observed in position F1 (i.e. at the SEOP cell outlet; Fig. 2a) and a pH change of +1 was found in position F2 (following extraction–compression) after 30 production cycles. Therefore it can be concluded that most of the Rb vapor condensed within the transfer lines and inside the extraction–compression unit. The preliminary finding suggest that physiologically harmful pH changes in rodent lungs after a few cryogenics-free hp gas deliveries are not likely, even with the high Rb density at ^83^Kr SEOP conditions and in the absence of gas filters. Although filter usage may still be prudent for further reducing any potentially remaining Rb contamination, a study detailing the exact quantity of the Rb carried through the gas extraction process and the effects of filtering techniques upon the spin polarization is beyond the scope of this work.

### 4.4 Mixing of hp ^129^Xe or hp ^83^Kr with O_2_ during extraction

**Fig. 7:**
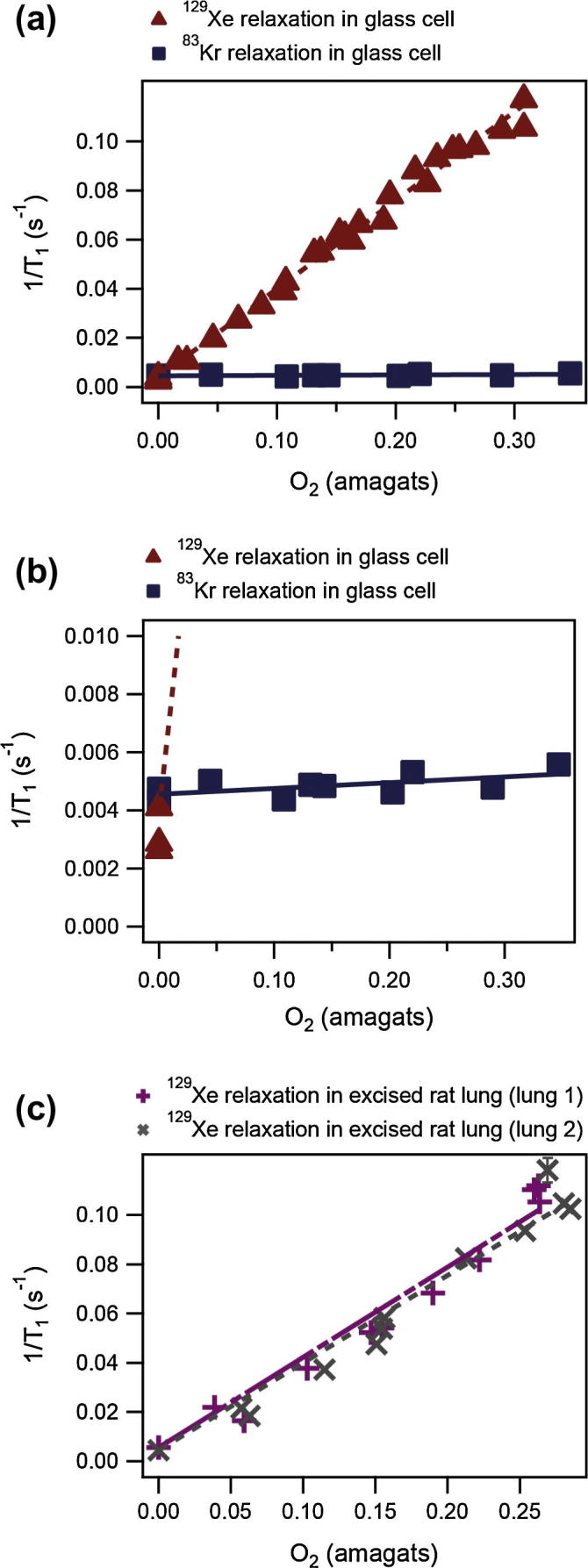
Relaxation rate (1/*T*_1_) of hp noble gas as a function of O_2_ density. Hyperpolarized noble gas–N_2_ mixtures were mixed at low pressure with O_2_ using the piston extraction unit. Hp gas was delivered into detection cell similar to Extraction Scheme 2. (a) ^129^Xe and ^83^Kr bulk gas phase relaxation measurements in a detection cell. Note that the relaxation rate depends linearly on O_2_ density for both isotopes. ^129^Xe data agreed well with literature values [31]. (b) A zoom in into the intercept area of (a). Note that ^83^Kr relaxation rate was more than 2 orders of magnitude less dependent on O_2_ density than that of ^129^Xe. Also note the crossover of the Xe and Kr data, further explanation are in the text. (c) ^129^Xe relaxation rate as a function of O_2_ density inside two excised rat lungs. ^129^Xe relaxation rate dependence on O_2_ density remained the same for the lungs studied. The average slope for the two data sets of $1/T_{1}\rho_{{\text{O}}_{2}}=0.361\;\pm \;0.020\;{\text{s}}^{-1}\;{\text{amagat}}^{-1}$ compared well with $1/T_{1}\rho_{{\text{O}}_{2}}=0.360\;\pm \;0.007\;{\text{s}}^{-1}\;{\text{amagat}}^{-1}$ determined in the glass detection cell. Note that the relaxation rate of ^129^Xe in a lung in the absence of O_2_ was $1/T_{1}^{\left(0\right)}=5.0\;\pm \;0.5\;\times \;10^{-3}\;{\text{s}}^{-1}$. The error bars represent the systematic error of the experiment.

Extraction scheme 2 was modified to generate hp gas mixtures with a precisely selected O_2_ concentration. After transfer of the hp gas into the volume $V_{\mathrm{ext}}^{\max}$ of the extraction unit, O_2_ was added and resulted in a carefully regulated pressure increase until the desired O_2_ concentration was reached. The total pressure in the large volume $V_{\mathrm{ext}}^{\max}$ = 790 ml was typically between 10–20 kPa and the mixing of the gasses was sufficient within 5 s after addition of O_2_. The method was tested by measuring the ^129^Xe longitudinal relaxation rates caused by paramagnetic O_2_ as a function of O_2_ density (or corresponding oxygen concentration; shown in Fig. 7).

The O_2_ density dependent relaxation data shown in Fig. 7a (filled triangles) demonstrated the accuracy in the preparation of the gas mixture. The data was obtained using a series of small flip angle pulses at physiologically relevant, (i.e. ambient) pressure. The resulting slope of the oxygen density dependent ^129^Xe relaxation rate

(2) $${\left(\frac{1}{T_{1}\rho_{{\text{O}}_{2}}}\right)}_{{}^{129}\text{Xe}}^{290\;\text{K,}9.4\;\text{T}}=0.360\;\pm \;0.007\;{\text{s}}^{-1}\;{\text{amagat}}^{-1}$$

at 9.4 T field strength and 290 K was in good agreement with that obtained by Jameson et al. with thermally polarized ^129^Xe at high xenon and oxygen densities [31]:

(3) $${\left(\frac{1}{T_{1}\rho_{{\text{O}}_{2}}}\right)}_{{}^{129}\text{Xe}}^{9.4\;\text{T}}=0.343\;{\text{s}}^{-1}\;{\text{amagat}}^{1}\cdot {\left(T/300\;\text{K}\right)}^{-0.03}$$

where *T* is the temperature of the gas mixture in Kelvin. An amagat is defined in this work as the density of an ideal gas at standard pressure and temperature of 101.325 kPa and 273.15 K and therefore $1\;\text{amagat}=2.6868\times 10^{25}\;{\text{m}}^{-3}$. At the conditions used in this work, N_2_, O_2_, Kr, and Xe are considered to follow ideal gas laws. According to Eq. (2), a relaxation time of *T*_1_ = 14.2 s was observed for a 21% O_2_, 79% hp ^129^Xe–N_2_ mixture contained in an NMR test tube at 9.4 T and ambient pressure. However, the experimental setup used in this work was also applied to relaxation measurements in lungs as shown in Fig. 7c after SEOP, hp gas extraction, mixing with a quantified amount of O_2_, compression, transfer into a storage container, and inhalation by the excised lungs. The average longitudinal relaxation rate for two excised lungs was found to have the following dependence:

(4) $${\left(\frac{1}{T_{1}\rho_{{\text{O}}_{2}}}\right)}_{{}^{129}\text{Xe}}^{290\;\text{K,}9.4\;\text{T}}=0.361\pm 0.020\;{\text{s}}^{-1}\;{\text{amagat}}^{-1}$$

Eq. (4) describes the oxygen dependent term of the ^129^Xe *T*_1_ relaxation, however the average longitudinal relaxation rate measured in the absence of oxygen (i.e. the zero density intercept in Fig. 7a) was $1/T_{1}^{\left(0\right)}=5.0\pm 0.5\times 10^{-3}\;{\text{s}}^{-1}$ in the two lungs. Neglecting the very small contribution of ^129^Xe gas phase interactions to the longitudinal relaxation, the oxygen independent term in the lung is essentially relaxation caused by relaxation of tissue-dissolved xenon that is in rapid exchange with the gas phase. The average slope of the oxygen density dependent relaxation for the two rat lungs is in good agreement with Eq. (2). This agreement indicates that the presence of the excised lung did not strongly affect the hp ^129^Xe relaxation dependence on oxygen (i.e. compared to the bulk gas phase), despite tissue dissolved O_2_ and approximately 1–2% tissue dissolved xenon [32]. In any case, Extraction Scheme 2 enabled precise mixing of O_2_ with the hp gas during the extraction process and thus may be of use for future hp ^129^Xe measurements of *in vivo* oxygen partial pressures that provide lung functional information about oxygen exchange in lungs [33].

The effect of paramagnetic oxygen upon the ^83^Kr relaxation behavior is shown in Fig. 7a and b. The oxygen density dependent ^83^Kr relaxation rates exhibited a slope that is approximately two orders of magnitude smaller than that for ^129^Xe:

(5) $${\left(\frac{1}{T_{1}\rho_{{\text{O}}_{2}}}\right)}_{{}^{83}\text{Kr,}(25\%\text{Kr,}75\%{\text{N}}_{2})}^{290\;\text{K,}9.4\;\text{T}}=0.002\pm 0.0009\;{\text{s}}^{-1}\;{\text{amagat}}^{-1}$$

The vast difference in observed relaxation behavior between xenon and krypton due to the presence of paramagnetic oxygen were mostly caused by the difference in the square of the gyromagnetic ratios ${\left(\gamma_{I}\right)}_{{}^{129}\mathrm{Xe}}^{2}/{\left(\gamma_{I}\right)}_{{}^{83}\mathrm{Kr}}^{2}\approx 51.9$ [34]. However, unlike the ^129^Xe–O_2_ pair [31] or the ^3^He–O_2_ interaction [35], the situation for ^83^Kr is complicated by quadrupolar relaxation that makes quantitative interpretation of the paramagnetic contributions difficult. As can be seen from the (zero oxygen density) intercept in Fig. 7b, quadrupolar relaxation of gaseous ^83^Kr in a macroscopic container dominated over the paramagnetic contributions to the relaxation, at least for the investigated O_2_ concentrations. Quadrupolar relaxation $\left(T_{1}^{Q}\right)$ arises from surface interactions [36], gas composition dependent van der Waals complexes, and gas pressure and composition dependent binary collisions [37,38]; as shown in following equation:

(6) $$\frac{1}{T_{1}}=\frac{1}{T_{1}^{\mathrm{para}}}+\frac{1}{T_{1}^{\mathrm{surface}}}+\frac{1}{T_{1}^{\mathrm{vdW}}}+\frac{1}{T_{1}^{\mathrm{binary}}}$$

Due to quadrupolar relaxation, Eq. (5) is only valid for O_2_ added to the particular 25% krypton–75% N_2_ mixture because different krypton–nitrogen ratios will result to different ${\left(1/T_{1}\rho_{{\text{O}}_{2}}\right)}_{{}^{83}\text{Kr}}$ values. Note that quadrupolar relaxation dominated over paramagnetic relaxation even in the macroscopic gas container with small S/V and concentrations of up to 40% O_2_. It should therefore come at no surprise that similar O_2_ concentrations did not affect the ^83^Kr relaxation in rat lungs where high S/V lead to $T_{1}\approx 1-1.2\;\text{s}$ [15].

## 5 Conclusions

Cryogenics free hp ^129^Xe and hp ^83^Kr production is feasible for biomedical MRI applications. The methodology is based on previous results that found high apparent spin polarization values *P_app_* at high xenon (or krypton) concentrations in low-pressure SEOP using line narrowed diode array laser. In this work, focused on the cryogenics free hp gas extraction and transfer steps, the maximum apparent polarization of the noble gas was found to be $P_{\mathrm{app}}\approx 14\%$ for ^129^Xe and approximately *P_app_* = 3.5% for ^83^Kr using only 23.3 W laser power incident at the SEOP cell. The volume of the hp gas was ∼18 ml after 6 min SEOP for hp ^129^Xe and ∼34 ml after 8 min SEOP for hp ^83^Kr. The explored methodology was based on stopped flow SEOP and larger volumes per unit time require either the usage of larger SEOP cells and higher laser power. Alternatively, many SEOP units can be run in parallel. Furthermore, polarization can be further improved through higher than the 23.3 W of laser power used in this work.

Simple pH based tests indicated on the minimal rubidium content of the ambient pressure hp gas to be minimal despite the absence of gas filters used during the hp gas extraction. The presented methodology therefore allows for a simplified and, with higher laser power becoming more readily available, potentially low cost hp ^129^Xe production method. The generated polarization *P_app_* and the volume of hp gas were sufficient for slice selective, coronal hp ^129^Xe MR images of excised rodent lungs in a single inhalation cycle. The methodology is crucial for hp ^83^Kr MRI and single inhalation cycle images using isotopically enriched ^83^Kr were obtained. An extraction scheme utilizing a single cycle piston pump was shown to accomplish efficient hp ^83^Kr gas extraction that preserved *P_app_* at a high level. In comparison a much simpler inflatable balloon based extraction scheme was found to be remarkably efficient for hp ^129^Xe extraction.

For both noble gases, the piston pump based extraction scheme allowed for precise mixing of the hp gas with a selected quantity of oxygen. This procedure may be helpful for *in vivo* studies, such as oxygen partial pressure measurements in lungs. Excised lung data suggests that the ^129^Xe *T*_1_ relaxation dependence on the O_2_ concentration is very similar to that found in the bulk gas phase. In the absence of O_2_, the ^129^Xe *T*_1_ relaxation within the excised lungs was $T_{1}^{\left(0\right)}=200\pm 20\;\text{s}$. Furthermore, the method enabled the first quantitative bulk gas phase measurement of ^83^Kr longitudinal relaxation as a function of O_2_ concentration. It was found that ^83^Kr is approximately two orders of magnitude less sensitive to the presence of O_2_ than ^129^Xe.

## 6 Materials and methods

### 6.1 Optical pumping of ^129^Xe and ^83^Kr

The low-pressure batch-mode Rb-SEOP method used in these experiments is similar to the one described in detail in Ref. [10]. In this work, 23.3 W of circularly polarized laser light was incident at the front of the SEOP cell (Fig. 2a). All gases used were research grade: Kr (99.995% pure; natural abundance, 11.5% ^83^Kr; Airgas, Rednor, PA, USA), Xe (99.995% pure; natural abundance, 26.4% ^129^Xe; Nova Gas Technologies, Charleston, SC, USA), isotopically enriched Kr (enriched to 99.925% ^83^Kr; Chemgas, Boulogne, France), isotopically enriched Xe (99.995% pure; enriched to 83% ^129^Xe; Nova Gas Technologies, Charleston, SC, USA), and N_2_ (99.999% pure; Air Liquide, Coleshill, UK).

To ensure the quality of the SEOP cell at least four polarization measurements of a standard mixture (5% Xe–95% N_2_ or 25% Kr–75% N_2_ for xenon or krypton experiments respectively) were acquired using an SEOP cell pressure of 230 ± 20 kPa before starting experiments. If a polarization of less than 40% is observed for Xe or 3.5% for Kr then the SEOP cell is replaced. To verify the SEOP cell performance and attempt to prevent polarization fluctuations from affecting the observed functional relationship, polarization values at high SEOP cell pressure are taken at least four times at irregular intervals during the experiment.

### 6.2 Extraction units

For Extraction Scheme 1, a pressure chamber was constructed from an acrylic tube (length: 200 mm, inner diameter: 100 mm). As shown in Fig. 2b, a latex balloon was placed inside the pressure chamber and was connected to an acrylic screw cap that sealed the body of the chamber (vacuum tested to 0.1 kPa, pressure tested to 300 kPa). The internal volume of the balloon was connected to valve (A) through the screw cap via a small channel with minimized internal volume.

For Extraction Scheme 2, a large volume piston pump unit was constructed from an acrylic tube (length: 450 mm, inner diameter: 58 mm, outer diameter: 70 mm) with acrylic screw caps attached to the tubing and that were each fitted with an O-ring that sealed the device (vacuum tested to 0.1 kPa, pressure tested to 300 kPa). The extraction unit was encompassed by a solenoid coil that produced a static magnetic field of *B*_0_ = 0.005 T that aimed to reduce the relaxation of the hp ^83^Kr inside the extraction unit [36]. The extraction unit needed to attain vacuum conditions of less than 0.2 kPa prior to hp gas extraction from the SEOP cell and then compress the extracted hp gas to ambient pressure. An O-ring seal equipped acrylic piston provided gas tight isolation of the two compartments of the extraction unit. The length of the piston was 150 mm to provide proper alignment but its particular shape, shown in Fig. 3a, reduced its weight to minimize its inertia.

Extraction Scheme 2 required multiple steps as described in Fig. 3b–e. Initially the piston was retracted by pressurizing *V_ext_* with N_2_ while simultaneously pulling a vacuum on the back of the piston (Fig. 3b). With the piston in its retracted position, the extraction volume of the unit was $V_{\mathrm{ext}}^{\max}=790\;{\text{cm}}^{3}$ and this volume was evacuated to below 0.2 kPa (Fig. 3c). $V_{\mathrm{ext}}$ is subsequently opened to the SEOP cell to allow for gas transfer from the SEOP cell (Fig. 3d). After 5 s, a pressure of approximately 6–13 kPa was reached (depending on the SEOP pressure), however the pressure equalization was only about 80% complete, allowing for a transfer of approximately 3/4 of the hp gas from the SEOP cell. The incomplete transfer of hp gas was deemed acceptable to limit relaxation in the chamber and gas lines. Hp gas was compressed by pressurizing the piston with >100 kPa of N_2_, leading to the scenario depicted in Fig. 3e. The extraction–compression unit was then opened to either a detection cell for polarization measurements or to the storage volume (*V_B_*) for lung MRI.

### 6.3 Spectroscopic experiments

Polarization measurements and *T*_1_ relaxation of either hp gas–O_2_ mixtures in a bulk gas phase were conducted in a vertical bore 9.4 T superconducting magnet (Oxford Instruments, UK) equipped with a Magritek Kea 2 spectrometer (Wellington, New Zealand) using 15 mm custom build probes tuned to the resonance frequency of ^129^Xe (110.56 MHz) and of ^83^Kr (15.38 MHz). *T*_1_ relaxation measurements in the excised lung were performed in a vertical bore 9.4 T Bruker Avance III microimaging system using a 25 mm ^129^Xe custom build birdcage probe tuned to 110.69 MHz.

### 6.4 Imaging protocol

MRI experiments were performed in a vertical bore 9.4 T Bruker Avance III microimaging system. A custom build 25 mm birdcage probe tuned to 110.69 MHz and a commercial 30 mm probe (Bruker Corporation, Billerica, Massachusetts, USA) tuned to 15.40 MHz were used for ^129^Xe or ^83^Kr imaging experiments, respectively.

^129^Xe images were acquired using a variable flip angle (VFA) FLASH sequence [29] using 64 gradient increments in phase-encoding dimension resulting in a total image acquisition time of 13.8 s. The resulting data size was 128 × 64 with the field of view (FOV) of 46.9 × 30.0 mm^2^ in the frequency encoding and in the phase encoding dimensions, respectively. An MRI image of a 4 mm central slice of the lung in coronal orientation was obtained using sinc-shaped pulses with 1 ms in length and a variable amplitude for each phase encoding gradient increment. A subsequent non-slice selective image was obtained using rectangular pulses with variable amplitudes during the same inhalation cycle.

^83^Kr image data were collected using VFA FLASH sequence with 32 phase encoding gradient increments resulting in the final data size of 64 × 32. Variable amplitude 0.8 ms gaussian pulses or 2.0 ms sinc-shaped pulses were used in image acquisition. The total acquisition time was either 0.57 s or 0.62 s depending on the length of the used excitation pulse. The resulting image length was either 51.0 mm or 50.9 mm in the frequency encoding and 38.1 mm or 40.7 mm in the phase-encoding dimension, respectively.

Data were processed using Prospa (v. 3.06; Magritek, New Zealand). The data were apodized in both dimensions using sine-bell squared function prior to the image reconstruction further image processing and analysis were performed with IGOR Pro (v 6.11, Wavemetrics, USA).

### 6.5 Animal care and preparation

**Fig. 8:**
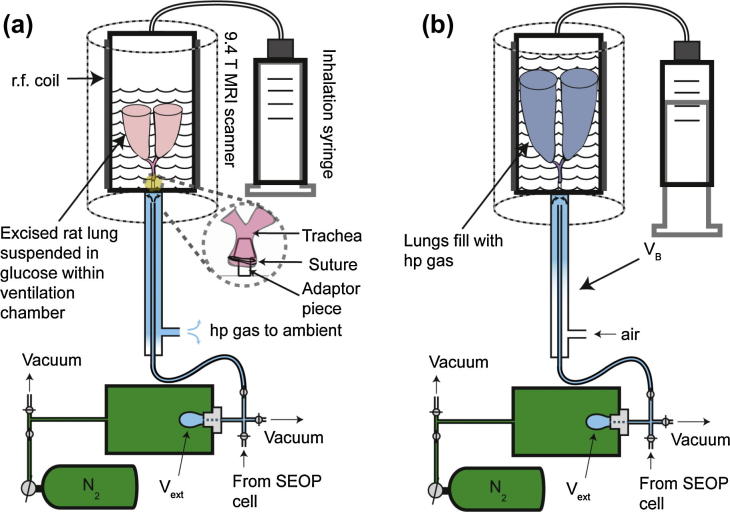
Hp gas delivery to the lung ventilation chamber using the ambient pressure storage volume (*V_B_*). Note that Extraction Scheme 2 uses the same methodology. (a) Hp gas is driven from *V_ext_* into the storage volume (*V_B_*) displacing the existing gas in *V_B_* that exhausts to ambient. (b) The inhalation syringe is pulled to produce a slight vacuum on the glucose solution, forcing the lung to expand and to uptake hp gas from *V_B_*.

Male Sprague–Dawley rats (Charles River, Margate, UK) weighing 360–450 g were used in this study. These weights of rat were chosen as they roughly corresponded to the maximum lung size that would fit into the ventilation chamber (Fig. 8). Rats were humanely euthanized by overdose of pentobarbital (Sigma–Aldrich Ltd., Gillingham, UK) in accordance with A(SP)A 1986 (Animals for Scientific Procedures Act 1986) and local animal welfare guidelines. Once death was confirmed the pulmonary system was flushed with a heparin-solution (Wockhardt UK Ltd., Wrexham, UK) via catheter inserted into the right ventricle or caudal vena cava. This was followed by Dublecco’s phosphate buffer solution (D-PBS, Sigma–Aldrich Ltd., Gillingham, UK) to remove remaining blood from circulation. The lungs were inflated with around 3 ml of air and the trachea clamped; then the lungs, heart, and connective tissue were extracted *en bloc*. After extraction the lung’s trachea was cannulated and a syringe was used to breathe the lungs to ensure that they did not leak. Lungs were stored in glucose solution (5% glucose in water, Baxter Healthcare Ltd., Thetford, UK), chilled to approximately 280 K until needed.

### 6.6 Lung ventilation and gas delivery

Excised rat lungs were inserted into a custom-made, sealable, ventilation chamber that filled the entire coil region. The ventilation chamber and its operating procedures are described in detail in previous work [15]. Briefly, the trachea of the rat lung was cannulated with an adaptor that was attached to the top of the ventilation chamber. The ventilation chamber was filled to about 2/3 of its total volume with a 5% glucose solution (Baxter Healthcare Ltd., Thetford, UK). Hp gas was delivered to the storage volume *V_B_* after compression using one of the two Extraction Schemes described in this work. When a volume was pulled on the inhalation syringe pressure equalization forces the lungs to expand (Fig. 8). This acts in a similar fashion to the thoracic diaphragm, as the expansion of the lungs causes it to inhale gas from the volume *V_B_*.

### 6.7 Rubidium filters

Rubidium filters were made from 60 mm of Teflon tubing (outer-diameter = 9.4 mm, inner-diameter = 6.4 mm; Swagelok, Warrington, UK) with 100 g of glass wool (Corning glass works, Corning, NY, USA) loosely packed inside. Chemical indicator paper (Whatman plc, Maidstone, UK) was used to check the pH value of the 1.0 ml of distilled water used to wash the glass wool. The resulting pH of the rubidium wash was pH 5.0.

### 6.8 O_2_ mixing with hp gas and relaxation measurements

After SEOP at 220 kPa, a transfer of 5 s in duration resulted in a pressure of approximately 11 kPa of hp gas in *V_ext_*. Valves A + B (Fig. 3a) were closed and the connecting lines were evacuated. A selected pressure of O_2_ gas was then added to *V_ext_* and the connecting lines were evacuated again. After a 5 s time delay that allowed for mixing of the O_2_ with the hp gas, the mixture was delivered for the MR measurements performed using Extraction Scheme 2.

All *T*_1_ data were obtained at ambient temperature using a pulse sequence comprising of sixteen medium (θ=12°) flip angle r.f. pulses evenly separated by time increment τ. *T*_1_ relaxation values were determined from the nonlinear least-square analysis of the time dependence of the NMR signal intensity *f*(*t*) in the presence of spin-destruction due to the r.f. pulses [39] according to:

(7) $$f(t)=\cos(\theta^{t/\tau })e^{-t/T_{1}}$$

## Footnotes

3 Normalized polarization values slightly above unity are observed at higher SEOP cell pressures. This is likely caused by an inhomogeneous distribution of the hp gas within the SEOP cell that is only partially probed by the Baseline Scheme.
